# Improving the thermal stability of phytase using core-shell hydrogel beads

**DOI:** 10.1016/j.fochx.2023.101082

**Published:** 2023-12-16

**Authors:** Eunhye Yang, Hongmin Dong, Waritsara Khongkomolsakul, Younas Dadmohammadi, Alireza Abbaspourrad

**Affiliations:** Department of Food Science, College of Agriculture & Life Sciences, Cornell University, Stocking Hall, Ithaca, NY 14853, United States

**Keywords:** Core-shell hydrogel, Chitosan, Alginate, κ-Carrageenan, Phytase, Thermal Stability

## Abstract

A core–shell hydrogel bead system was designed to maintain the catalytic activity of phytase and protect its enzymatic functionality from heat treatment. The designed structure consists of a chitosan-phytase complex core and an alginate-carrageenan hydrogel shell. The core–shell hydrogel was optimized to improve phytase encapsulation efficiency and increase the thermal stability of the encapsulated phytase. After heat treatment, encapsulated phytase retained ∼ 70 % of its catalytic activity and the same secondary structure of free phytase. Fourier transform infrared spectroscopy indicated strong intermolecular interactions between chitosan and phytase in the core, but little interaction between the core and the alginate and κ-carrageenan shell, this supports the structural and functional stability of the phytase. Differential scanning calorimetry confirmed that the designed core–shell structure had a higher melting point. Encapsulating phytase in a core–shell hydrogel bead can enhance the thermal stability of phytase, which broadens the potential applications for phytase delivery.

## Highlights

• Core-shell hydrogel bead protects the catalytic activity of phytase from heat.
• The bead has a chitosan-phytase core and an alginate-carrageenan hydrogel shell.
• Encapsulated phytase retained 70% of its catalytic activity after heating at 90 ℃.
• Strong intermolecular interactions were observed between chitosan and phytase.
• Little interaction between the core and the alginate and κ-carrageenan shell.

## Graphical abstract

**Figure:**
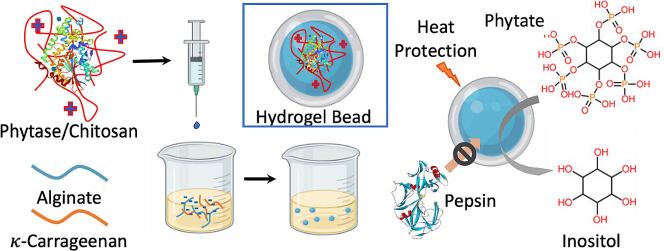

## 1 Introduction

Plant-based diets that are high in seeds and cereal grains contain a high level of phytate. Phytate is known to chelate Ca, Fe, Zn, and other minerals, which can lead to an increased risk of nutritional deficiencies in these essential minerals. Phytate chelates these minerals before they can be absorbed by the small intestine, exacerbating conditions such as iron deficiency (Sandberg & Scheers, 2016). The enzyme phytase catalyzes phytate hydrolysis, however, phytase in the digestive tract doesn't act on the phytate until it is too late, after the minerals have already been complexed (Rizwanuddin et al., 2023). Supplementing phytase into foods where it can act earlier on phytate could help avoid chelating minerals and improve the delivery of essential elements.

Unfortunately, foods cannot be directly fortified with phytase because it is neither stable to the high cooking temperatures nor resistant to the low pH of the digestive tract. Like other proteins or enzymes, phytase’s catalytic activity and structural stability are vulnerable to environmental stress. Specifically, when phytase is exposed to heat treatment at 95 °C, its activity decreases to about 4 % of untreated phytase (Weng et al., 2022). Also, it was reported to have less than 50 % phytase activity after in vitro digestion because of the vulnerability of phytases to proteolytic cleavage by pepsin (Menezes-Blackburn et al., 2015).

Several studies have tried to solve these problems using encapsulation systems (Weng et al., 2022). The encapsulation system of phytase for broad food-based applications requires the use of a food-grade biopolymer. Although many carriers have been investigated to encapsulate phytase, most are inorganic, such as zeolite and allophonic nano-clay, which are used for plant or animal feed applications and are either non-food grade or unsuitable for human consumption (Filippovich et al., 2023). A few studies investigated the use of natural food biopolymers to encapsulate phytase, for example, spray drying phytase with whey and guar gum was used to microencapsulate phytase (Garcia et al., 2019). About 90 % of the phytase activity was retained after simulated digestion in encapsulated phytase; however, the thermal stability of enzymes in the microcapsules was not significantly improved and thus could still not be used in hot food applications. Alginate-chitosan microcapsules to encapsulate phytase were also investigated (Vandenberg et al., 2011). Unfortunately, in this system, the alginate-chitosan microcapsules were too stable and were unable to release phytase even at the low pH levels of the gastric environment. The poor release of phytase decreased interactions between enzymes and phytates; therefore, a careful balance of heat-resistant polymers that can form proper interactions between polymer and phytase yet are capable of releasing phytase under the appropriate conditions is vital.

Based on recent studies (do Nascimento et al., 2022, Zhai et al., 2023), we expect that the core–shell structure can control this balance. A core–shell structure has various advantages, such as controlling the release speed, increasing drug efficiency, and enhancing the stability of the loaded materials. Various systems with core–shell structures have been developed, for example, hydrogel beads (do Nascimento et al., 2022), nanoparticles (Leena et al., 2022), and nanofibril (Huang et al., 2023).

Here, we report an enzyme encapsulation method for improving the heat resistance of phytase using a core–shell structure fabricated with polysaccharides. Chitosan, known for protecting enzyme activity and enhancing the thermal stability of therapeutic proteins (Li et al., 2022, Song et al., 2022, Xu and Liang, 2022) was chosen as the primary biopolymer to stabilize the phytase core. Alginate was selected because it immediately forms a solid gel network with calcium ions as cross-linkers (Tang et al., 2023). Further, when alginate is mixed with other polysaccharides, the stability of the alginate gel toward various stressors is improved (Bialas et al., 2021). We chose κ-carrageenan as a secondary polymer for the shell materials because it can absorb large amounts of heat and because of its heat-induced gelation characteristics (Liu & Li, 2016), which should ensure that the shell hardens after heat treatment and keeps the cargo from releasing. To make the core–shell hydrogel beads, drops of a phytase/chitosan mixture were added to the alginate/κ-carrageenan mixture and a core–shell hydrogel was formed on contact. The encapsulation efficiency and catalytic activity of phytase were assessed and different formulations investigated to optimize the beads. Then the physicochemical properties of the hydrogel beads were characterized, including core–shell structure, surface morphology, and intermolecular interaction. The effect of heat treatment was also studied by investigating thermal stability, release profile, secondary structure, and thermogram analysis.

## 2 Materials and methods

### 2.1 Materials

Potassium chloride (>99 %), alginic acid sodium salt from brown algae (medium viscosity), phytase from wheat (>0.01 unit/mg solid, 6-phytase), sodium hydroxide anhydrous (>97 %), calcium chloride dihydrate (>99.5 %), ammonium molybdate tetrahydrate, ethylene diamine tetra-acetic acid (EDTA) disodium salt (>99 %), trichloroacetic acid (TCA), fluorescein isothiocyanate isomer I (FITC), and l-ascorbic acid were purchased from Sigma-Aldrich (St. Louis, MO, US). Hydrochloric acid and Pierce^TM^ rapid gold BCA (bicinchoninic acid) protein assay kits were purchased from Thermo Fisher Scientific (Liverpool, NY, US). Pepsin (min. 3,000 units/mg) was purchased from Mallinckrodt Chemicals (Dublin, Ireland). Sodium phytate (95 %) was purchased from Astatech Inc. (Bristol, PA, US). κ-Carrageenan was purchased from TIC GUMS (Westchester, IL, US). Chitosan from *Aspergillus niger* was purchased from Sarchem Laboratories (Farmingdale, NJ, US). Sulfuric acid (95–98 %) was purchased from Fluka Honeywell (Charlotte, NC, US).

### 2.2 Preparation of core–shell hydrogel beads

Solutions of phytase (2.0, 3.0, and 4.0 w/v %) and chitosan (3.0, 4.0, and 5.0 w/v%) were prepared separately. As an example, a 4.0 w/v % chitosan was prepared by dissolving 0.4 g of chitosan into 10 mL of distilled water with stirring overnight at room temperature (∼25 ℃) and then the pH was adjusted to pH 5.0 using 0.2 M NaOH solution. To make the complex between chitosan and phytase, phytase was added to the chitosan solutions; 0.2 g of phytase was added to the 4 w/v % chitosan solution, which resulted in a 2.0 w/v % concentration of phytase. The solution was stirred for 1 h. CaCl_2_ and KCl, the cross-linkers for the alginate and κ-carrageenan, were added to the chitosan and phytase mixture. As an example, 0.294 g (2 mmol) CaCl_2_ and 0.037 g (0.5 mmol) KCl were added to 10 mL of a phytase and chitosan solution, followed by stirring for 30 min at room temperature.

Meanwhile, 1 w/v % solutions of sodium alginate and κ-carrageenan were prepared separately by adding 1.0 g of sodium alginate or 1.0 g of κ-carrageenan to 100 mL of distilled water with stirring and then heating overnight at 60 ℃. After stirring overnight and cooling at room temperature, the pH was adjusted to pH 10.0 using a 0.2 M NaOH solution.

To make the core–shell hydrogel beads, the alginate and κ-carrageenan solutions were mixed with different ratios from 4:1 to 1:4 (v:v), with stirring at 50 ℃ and then cooled to room temperature. Then, the mixture of phytase, chitosan, and cross-linkers was loaded into a syringe fitted with a 30-gauge needle and added one drop at a time into the alginate and κ-carrageenan solution. After addition, the mixture is centrifuged (6,700*xg*), and the resulting core–shell hydrogel beads are isolated from the supernatant and cooled at 4 ℃ for 1 h to stabilize.

### 2.3 Encapsulation efficiency

To determine the encapsulation efficiency of phytase, a Pierce rapid gold BCA protein assay kit was used to measure the protein concentration of free phytase separated from the core–shell beads (Steć et al., 2023). After centrifugation (6,700*xg* for 10 min) to isolate the core–shell hydrogel beads, the supernatant was then treated with a mixture of cupric sulfate and a copper chelator in a microplate well for 5 min at room temperature. The absorbance was then measured at 480 nm using a plate reader (SpectraMax iD3, Molecular Devices, CA, US) and calculated using a standard curve for bovine serum albumin (BSA). The encapsulation efficiency of phytase in the beads was calculated according to the following equation:

$$\mathrm{Encapsulation}\;efficiency\;\left(\%\right)=\frac{\mathrm{Total}\;added\;phytase-Unloaded\;amount\;of\;phytase}{\mathrm{Total}\;added\;phytase}\times 100$$

### 2.4 Phytase activity assay

The phytase activity in the hydrogel beads is determined according to the previously reported method with some modifications (Duru Kamaci & Peksel, 2021). In short, the substrate solution (0.44 mM sodium phytate, pH 5.0, 0.1 M sodium acetate buffer) is heated for 10 min at 50 °C in a water bath. A phytase sample (three beads) is added to the substrate solution. The enzymatic reaction is conducted for 30 min at 50 °C and quenched by adding the denaturing agent (400 μL of 15 w/v% trichloroacetic acid (TCA) solution). After cooling at room temperature for 10 min, the solution is centrifuged at 6,700*xg* for 10 min, and the supernatant (100 μL) is collected. This supernatant is mixed with 1 mL of the color reagent (1:1:3 (v/v/v); 10 w/v% ascorbic acid: 2.5 w/v% ammonium molybdate: 1 M sulfuric acid) and 900 μL of distilled water, then heated for 15 min at 50 °C. After cooling at room temperature for 10 min, the absorbance was recorded at 820 nm via a UV–Vis spectrophotometer (UV-2600, Shimadzu, Kyoto, JP) to determine the enzymatic activity of phytase.

### 2.5 Physicochemical characterization of phytase-loaded core–shell hydrogel beads

#### 2.5.1 Scanning electron microscope (SEM)

The pore size and surface morphology of core–shell hydrogel beads were observed using a Zeiss Gemini 500 scanning electron microscope (Zeiss, DE). Samples were placed on the carbon-taped stub and coated with gold using a sputter coater (Denton Desk V, NJ, US). The coated samples are scanned and captured by a high-efficiency secondary electron detector with a 20.0 μm aperture (accelerating voltage = 1 kV).

#### 2.5.2 Fluorescence microscope

To observe the core–shell structure cross-sectionally, fluorescein isothiocyanate (FITC) was used to label the phytase before loading it into the beads. Fluorescence microscopy images of FITC-labeled phytase-loaded beads were taken at 10x with Leica DM IL LED microscope (Leica, Wetzlar, Germany) and AmScope digital camera MU1403 (AmScope, Irvine, CA, US).

#### 2.5.3 Circular dichroism (CD) spectroscopy

The secondary structure of the phytase was evaluated using CD spectroscopy. To the phytase-loaded beads, 0.2 M EDTA was added and the mixture incubated and shaken at room temperature overnight to release the phytase. The CD spectra of released phytase were measured using a JASCO-1500 circular dichroism spectrometer (JASCO, Easton, MD, US) at both far-UV and near-UV regions under constant nitrogen flush. The obtained data was converted to molar ellipticity, [*θ*] (deg cm^2^ dmol^−1^), using the DichroWeb online processing platform.

#### 2.5.4 Fourier transform infrared spectroscopy (FTIR)

Intermolecular interactions between the phytase, chitosan, alginate, and κ-carrageenan were investigated using FTIR spectroscopy. FTIR analysis was carried out on an IRAffinity-1S Spectrometer (Shimadzu Corporation, JP) fitted with a single-reflection attenuated total reflectance (ATR) accessory. The measurement was conducted with an average of 32 scans from 400 to 2000 cm^−1^ (resolution = 4 cm^−1^) after testing a background without sample.

#### 2.5.5 Differential scanning calorimetry (DSC)

To verify the heat-resistance of the core–shell hydrogel bead, thermal profiles of free phytase, encapsulated phytase, each composition of beads, were obtained under a nitrogen atmosphere (100 mL min^−1^) using Auto 2500 differential scanning calorimeter (TA Instruments, New Castle, DE, US). The samples were put into a *T*-zero aluminum pan and heated at 10 °C min^−1^ from 50 to 150 °C. The collected data was analyzed using Universal Analysis 2000 (ver. 4.5).

### 2.6 Statistical analysis

The obtained data were presented as means and standard deviations of triplicates and analyzed using Analysis of Variance (ANOVA). The differences between mean values were evaluated using the Tukey HSD comparison test (p < 0.05). All statistical analyses were performed using JMP Pro16 (SAS Institute, US) and plotted by GraphPad Prism10 (GraphPad Software Inc., US).

## 3 Results and discussion

### 3.1 Optimization of phytase-loaded core–shell hydrogel beads

#### 3.1.1 Initial pH adjustment

**Fig. 1:**
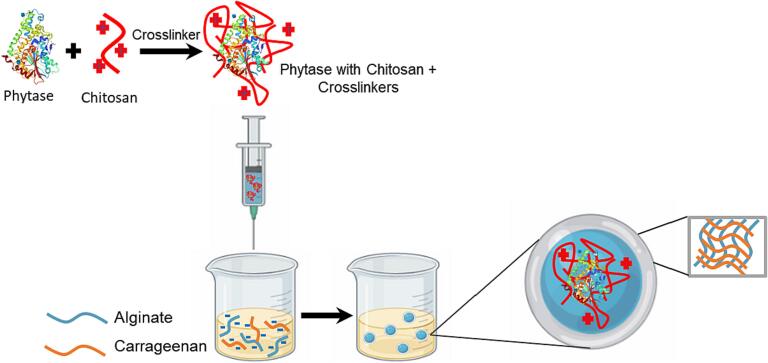
Schematic illustration of phytase-loaded core–shell hydrogel beads.

Chitosan was used as a core polymer, and a mixture of sodium alginate and κ-carrageenan served as the shell polymers, appearing in the schematic procedure of phytase-loaded core–shell hydrogel beads (Fig. 1). When the solution containing phytase, chitosan, and cross-linkers was dropped into the alginate and κ-carrageenan mixture, a core–shell structured hydrogel was formed with phytase in the core. The electrostatic interactions between the phytase and the chitosan form the core, and the diffused calcium ion cross-links the alginate around it to form the shell. Studies have shown that chitosan forms a complex with phytase at specific pH levels. Therefore, we optimized the pH range for our solutions to ensure good phytase-chitosan interactions (Ren et al., 2023).

At pH levels below its pKa, ∼6.5, chitosan has a positive charge, while phytase has a pI of 5.0, which means it carries an overall negative charge at higher pH levels. To induce a protein-polymer complexation by electrostatic attraction (Rao et al., 2009), the initial pH level of the chitosan solution was adjusted to pH 5 or pH 6. Within this range, a preliminary experiment confirmed that adding phytase to the chitosan solution raised the solution pH slightly (∼0.2 pH units); therefore, the initial pH of the chitosan solution was set to 5.0 to form the phytase/chitosan complex.

#### 3.1.2 The ratio of chitosan and phytase

The concentration of chitosan and phytase in the core must be optimized to make a stable core and improve the encapsulation efficiency of phytase. To optimize this aspect, we held both concentrations of calcium chloride and potassium chloride to 0.2 M and the alginate and κ-carrageenan both to 1 w/v %. Further, the chitosan and phytase complex needs to maintain an overall positive charge to electrostatically attract the alginate (contains carboxyl groups, pKa 3.5) and κ-carrageenan (contains sulfate groups, pKa 2.0), which both carry negative charges at higher pH levels (Gu et al., 2021). The zeta potential of the chitosan-complexed phytase and the encapsulation efficiency of phytase in the core–shell hydrogel beads were measured according to the concentration of chitosan and phytase.

**Fig. 2:**
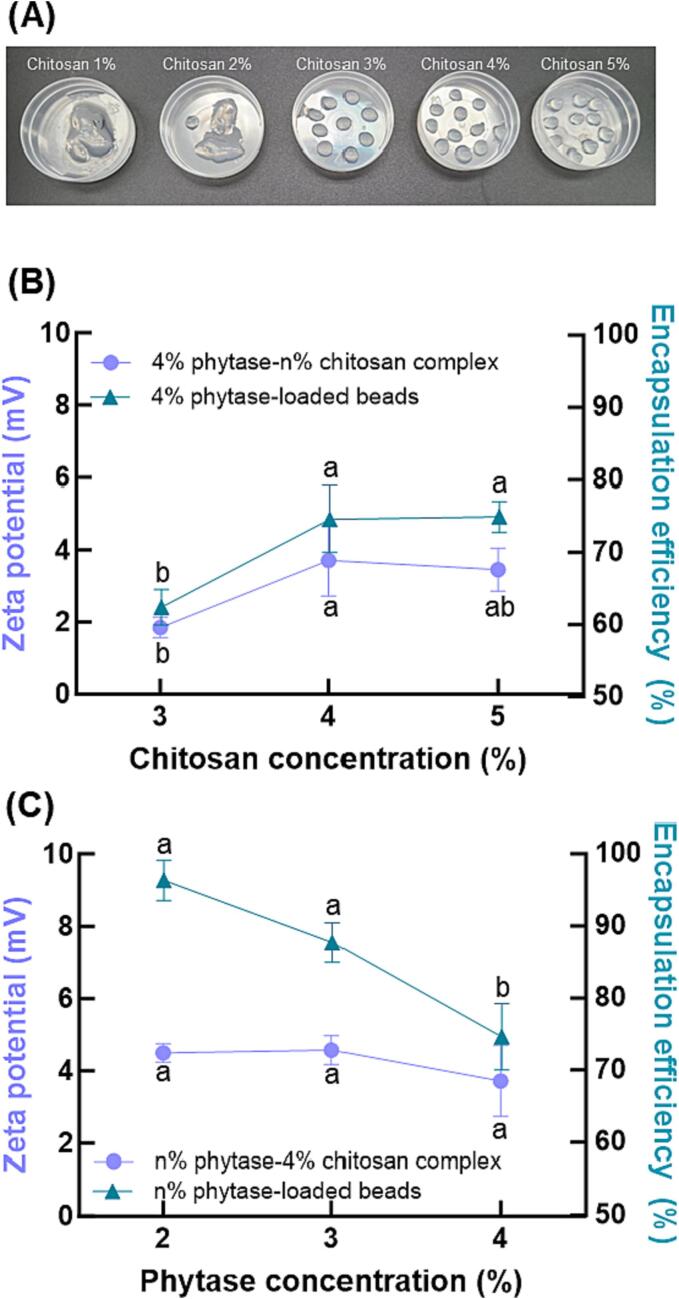
(A) Optical images of phytase-loaded core–shell hydrogel beads with different concentration of chitosan. Effect of (B) chitosan and (C) phytase concentration on zeta potential of phytase-chitosan complex at pH 5.0 and encapsulation efficiency of core–shell hydrogel beads including phytase-chitosan core and alginate-carrageenan shell.

The upper limit of the phytase concentration is 4 w/v % due to its solubility in the water at room temperature. Meanwhile, we found that the lower limit of the chitosan concentration is 3 w/v %, such that the chitosan and phytase solution have a viscosity suitable to form droplets that retain their shape and form the core of the core–shell structure (Fig. 2(A)). Even though the complex solution at all concentrations has a positive charge, the double-.

layer compression based on the surface charge screening by CaCl_2_ makes the zeta potential of the chitosan-phytase solution lower than the expected value (Du et al., 2005) (Fig. 2(B) and (C)).

Holding the phytase concentration at 4 w/v %, we found that adding 4 w/v % chitosan produced a complex with a high zeta potential (3.71 ± 0.99 mV) (Fig. 2(B)). However, core–shell hydrogel beads made using 2 w/v % phytase and 4 w/v % chitosan had the highest encapsulation efficiency of 74.60 ± 4.62 %. Conversely, when the chitosan concentration was fixed at 4 w/v%, the core–shell hydrogel beads made by 2 w/v % phytase had an encapsulation efficiency of 96.33 ± 2.78 % (Fig. 2(C)). Based on these results, the optimal conditions of the core were determined to be 4 w/v % chitosan and 2 w/v % phytase.

#### 3.1.3 Calcium chloride concentration

**Fig. 3:**
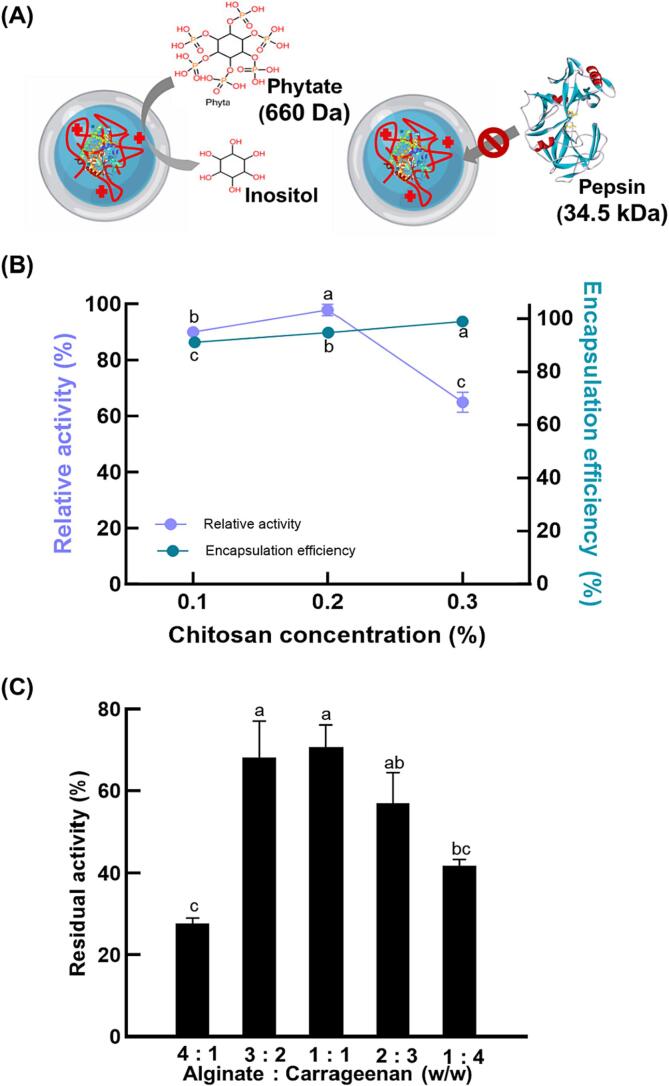
(A) Schematic illustration of core–shell hydrogel beads. (B) Effect of calcium chloride concentration on the relative activity of phytase-loaded core–shell hydrogel beads. (C) Effect of the ratio between alginate and carrageenan on the catalytic activity of phytase-loaded core–shell hydrogel beads after heat treatment at 90 °C for 10 min.

Following the optimization of the core, the shell requires two specific properties to be an effective phytase encapsulation system. First, it must allow phytate to diffuse across the shell to interact with phytase (molecular weight: 660 Da) and initiate phytase activity; and second, the shell needs to block the access of proteolytic enzymes, such as pepsin (molecular weight: 34.5 kDa), which could denature the phytase (Fig. 3(A)). According to a previous study, the lower the porosity of a shell network, the higher the diffusional resistance against proteolytic enzymes; however, this also slows down the diffusion of the substrate, in this case phytate, through the shell (McClements, 2017). Consequently, it is crucial to prepare a suitable shell with a sufficient level of cross-linking to improve encapsulation efficiency and maintain catalytic activity.

Compared with other factors, such as the concentration of biopolymers and incubation time, the diffusion rate of the Ca^2+^ ions is dependent on the CaCl_2_ concentration. To characterize the influence of calcium ion concentration on the shell, the core–shell beads were prepared with several different concentrations of calcium chloride, and then the phytase activity was measured keeping the concentrations of phytase at 2.0 w/v %; chitosan at 4 w/v %, alginate and κ-carrageenan at 1 w/v % each; and 0.05 M potassium chloride. Beads made with 0.2 M CaCl_2_ showed 97.87 ± 2.07 % activity, and for 0.1 M CaCl_2_, this activity decreased to 89.97 ± 1.85 %, while for 0.3 M CaCl_2_, the activity further decreased to 64.93 ± 3.58 % (Fig. 3(B)). At 0.1 M CaCl_2_, the shell is loose with a fragile cross-linked structure and while the phytate crosses into the core, there is some loss of activity due to lower encapsulation efficiency. Conversely, at 0.3 M CaCl_2_, while the concentration efficiency was similar to 0.2 M CaCl_2_, a more rigid shell was formed, and this firmer, thicker, and thus more compact network structure prevented the phytate from diffusing into the core and contacting the phytase (Qin et al., 2019). Accordingly, 0.2 M CaCl_2_ was the optimum concentration, maintaining almost all phytase activity.

At this point, we have identified that the optimized conditions include 2 w/v % phytase and 4 w/v % chitosan, 0.05 M KCl, and 0.2 M CaCl_2_; next we will optimize the ratio of alginate and κ-carrageenan.

#### 3.1.4 The ratio of alginate and κ-carrageenan

Alginate immediately forms a cross-linked solid gel network with calcium ions without heating (Tang et al., 2023), while κ-carrageenan prefers potassium ions as cross-linkers and incubation at 70 ℃, the gel hardens upon cooling to room temperature (Liu and Li, 2016, Lu et al., 2023, Yan et al., 2022). Due to these characteristics, alginate is expected to form the initial beads, and κ-carrageenan is expected to absorb heat during heating and form a harder gel through an endothermic process.

In addition, previous studies (Bialas et al., 2021) showed that when two or more types of polymers are mixed, gel stability against various stressors is improved. Mixing alginate and κ-carrageenan increased encapsulation efficiency by forming a more tightly packed biopolymer network structure. This tightly packed network results from the high number of carboxyl groups on sodium alginate and the hydroxyl groups on κ-carrageenan. These groups allow for intermolecular interactions between the biopolymers via hydrogen bonds and ionic interactions. These intermolecular interactions close the distance between the molecules of alginate and κ-carrageenan, encouraging the development of a tightly packed network structure (Yu et al., 2019).

The ratio of alginate and κ-carrageenan was optimized to improve the thermal stability of phytase and maintain encapsulation efficiency. The concentrations of all other materials were done at previously optimized levels. The core–shell beads were prepared with different ratios of alginate to κ-carrageenan from 4:1 to 1:4, and then the residual activity of phytase was measured after heat treatment at 90 °C for 10 min. The 1:1 ratio maintained the highest phytase activity, 70.67 ± 5.51 % (Fig. 3(C)). The higher the κ-carrageenan ratio, the higher the thermal stability because κ-carrageenan absorbs heat and forms a more durable shell. But, if the alginate ratio is too low, the entrapment of phytase is difficult because the initial cross-linking of the shell is based on the gelation of alginate. Based on these results, the 1:1 ratio was selected as the optimal condition for phytase-loaded core–shell hydrogel beads. Finally, we decided that the optimized conditions for the core–shell hydrogel formation that resulted in the best phytase activity and encapsulation efficiency were 2 w/v % phytase, 4 w/v % chitosan, 0.05 M KCl, and 0.2 M CaCl_2_, a 1:1 ratio of 1 w/v % of alginate and κ-carrageenan. Also, using the optimized samples, we measured the residual activity after heat treatment at 90 °C for 10 min and pepsin (2,500 U/mL, pH 2) treatment at 37 °C for 2 h. As a result, 62 % of the phytase activity was preserved, which we attribute to the large size and molecular weight of pepsin (34.5 kDa), making it difficult for pepsin to penetrate the core–shell beads.

### 3.2 Characterization of phytase-loaded core–shell hydrogel

#### 3.2.1 Morphology

**Fig. 4:**
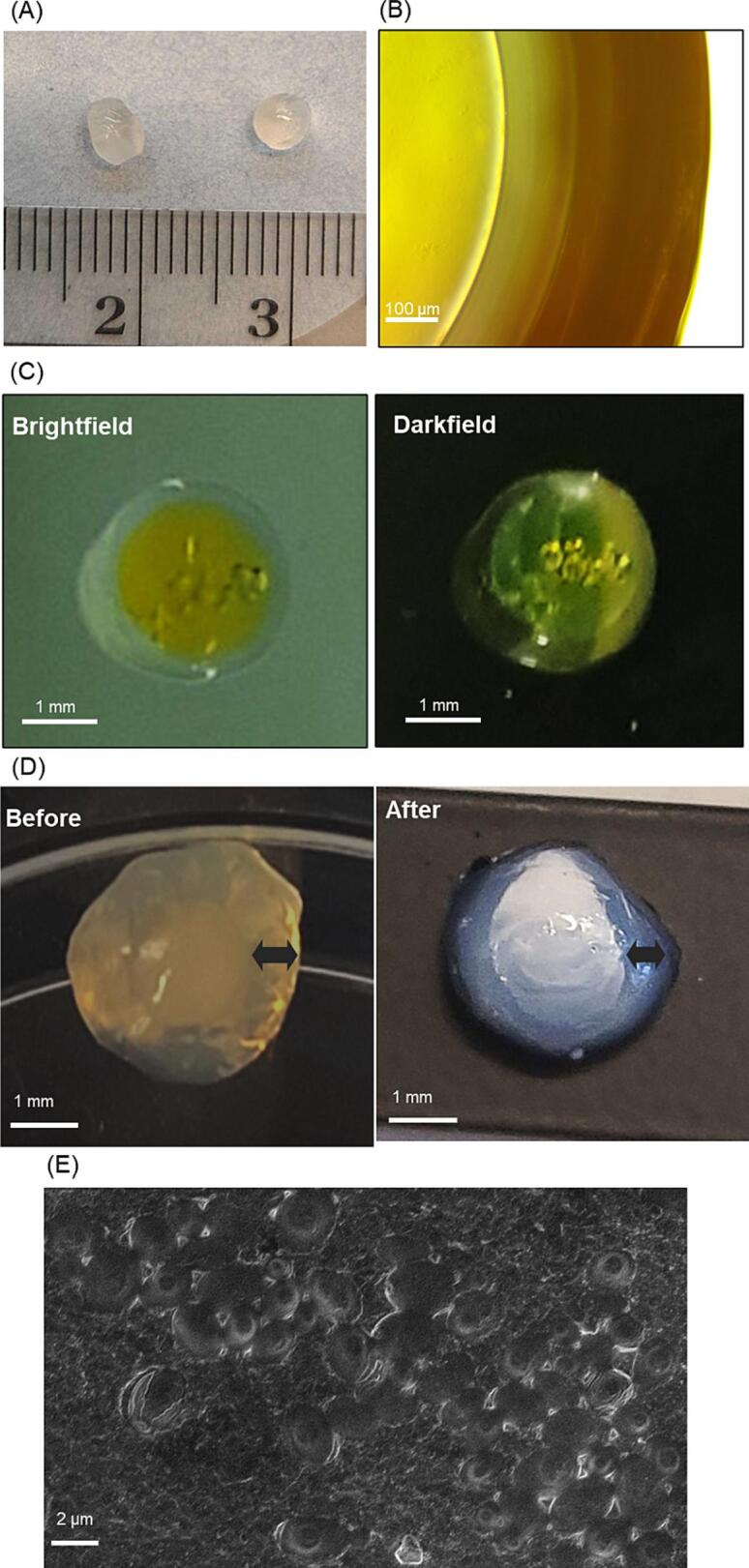
Morphological characterization of phytase-loaded core–shell hydrogel beads: (A) Optical images of phytase-loaded core–shell hydrogel beads. (B-C) Optical microscope images of phytase-loaded core–shell hydrogel beads after fluorescence labeling (scale bar = 100 μm, 1 mm). (D) Core-shell structure of the beads before and after heat treatment (scale bar = 1 mm). (E) SEM images of the surface on the core–shell hydrogel beads (scale bar = 2 μm).

The final optimized beads were prepared, and their surface structure was observed using optical microscopy and SEM. The optical image showed that the beads were spherical and opaque and had a uniform size with an average bead diameter of 3.7 ± 0.4 mm (Fig. 4(A)). To distinguish and observe the core and shell more clearly, the phytase was labeled with FITC, which is a widely used fluorescent probe for protein (Fig. 4(B) and (C)). The yellow-labeled cores appeared in both brightfield and darkfield. In addition, the shell with a thickness of 0.4 mm was observed when magnified with a microscope. Even without fluorescence labeling, a difference between the core of the beads and the shell of the beads can be seen in the optical images (Fig. 4(D)). After heat treatment for 10 min at 90 °C, there was little change in the appearance of the beads, suggesting that the shell, including alginate and κ-carrageenan, can survive heat stress. Although the core becomes opaque, this also occurred without phytase being present therefore, we believe this change is not related to the stability of the phytase chitosan mixture. Specifically, the gelation characteristic of κ-carrageenan, after heat and cooling, hardens the surrounding shell and strengthens the structure of core–shell hydrogel beads and leads to a more transparent and shell.

SEM analysis revealed the surface microstructure of the core–shell hydrogel beads (Fig. 4(E)). The porous structure of the core–shell hydrogel beads showed uniform macropores of approximately 1.1 ± 0.1 μm diameter. This is bigger than the expected size of a chitosan-phytase complex (∼100 nm), which could mean that the phytase might diffuse out of the bead; however, there are several reasons that a hydrogel with 1 μm pore size can prevent the dilution of phytase and the diffusion of pepsin into the bead while still allowing access to phytate. First there is electrostatic repulsion between the shell materials and pepsin: alginate.

and κ-carrageenan carry negative charges across the entire range of pH values; and pepsin (pI 3.2) is also expected to have a negative charge due to its pI value. Second, the diffusion index decreases rapidly with increasing molecular weight, making it more difficult for pepsin to diffuse into the bead, in contrast to smaller ions and molecules such as phytate (van den Berg et al., 2022). This mass and sized-based diffusion index also plays a role in lowering the release of complexed phytase from the beads. Because the phytase is complexed with chitosan in the core of the bead, the size of this complexed phytase-chitosan (natural phytase: 32.6 kDa) is much larger than phytase alone and, therefore, does not diffuse out of the bead easily. Finally, even if pepsin does diffuse into the core, it is expected that the encapsulated phytase will have improved stability against protease due to the complex it forms with chitosan.

#### 3.2.2 FTIR analysis of core–shell hydrogel beads

**Fig. 5:**
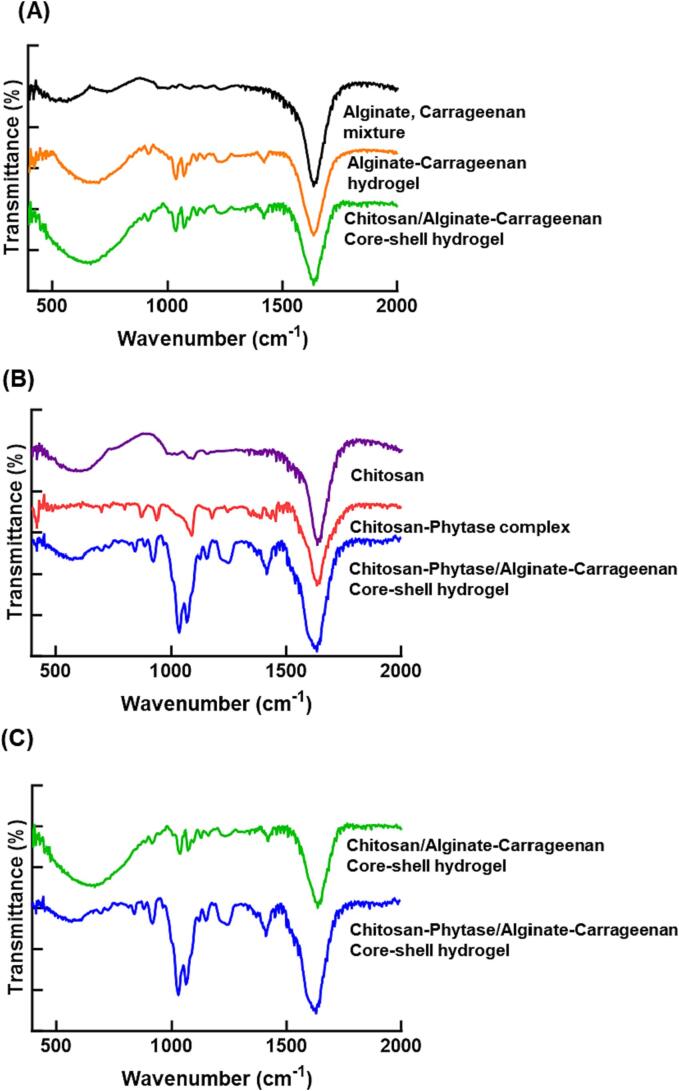
Molecular interaction between each composition of phytase-loaded core–shell hydrogel beads, using FT-IR analysis; (A) The mixture of alginate and carrageenan, the hydrogel including alginate and carrageenan, and the core–shell hydrogel including chitosan core and alginate-carrageenan shell. (B) Chitosan solution, the complex of chitosan and phytase, and the core–shell hydrogel including chitosan-phytase core and alginate-carrageenan shell. (C) The core–shell hydrogels including chitosan core and alginate-carrageenan shell with phytase and without phytase.

The interaction between each compound in the complexes and hydrogel beads was investigated through FTIR (Fig. 5). First, the alginate and κ-carrageenan mixture, alginate-κ-carrageenan hydrogel beads, and chitosan/alginate-κ-carrageenan beads were compared within 400–2000 cm^−1^ (Fig. 5(A)). To form an alginate-κ-carrageenan hydrogel, the bands at 1036 cm^−1^, 1094 cm^−1^, and 1234 cm^−1^ were related to the C—O stretching vibration of the C-OH of the primary alcohol. Moreover, a band at 1070 cm^−1^ was related to S=O stretching of the sulfoxide of κ-carrageenan. A band at 1420 cm^−1^ was related to asymmetrical stretching vibrations of the ionized –COO- group of alginates.

Meanwhile, there are two differences between alginate-κ-carrageenan beads and chitosan/alginate-κ-carrageenan beads; the bands shift from 673 cm^−1^ to 654 cm^−1^ with intensity increase and the peak shifted from 920 cm^−1^ to 916 cm^−1^. Because there is no critical bonding between the chitosan and the shell except for these small shifts, it suggests that the core and shell are maintained without significant interactions with each other. It indicates that chitosan is the only compound to interact with the phytase structure, so it is possible to control the tightness of interaction. Also, the shell is separated from the core and can act appropriately as a shield for stabilizing phytase.

The chitosan and chitosan-complexed phytase have different peaks, including 1100 cm^−1^ and 1200 cm^−1^ (Fig. 5(B)). A deformation in the vibration of the NH_2_ groups and CN stretching bands indicates that phytase was successfully immobilized in the chitosan-phytase complex. It is expected that the complexation based on these interactions can help the stabilization of phytase. Furthermore, compared with chitosan-complexed phytase and chitosan/alginate-κ-carrageenan beads, the bands of chitosan-phytase/alginate- κ-carrageenan beads shifted from 1100 cm^−1^, 1234 cm^−1^, and 1420 cm^−1^ to 1126 cm^−1^, 1248 cm^−1^ and 1418 cm^−1^, respectively (Fig. 5(C)). The gel structure based on these interactions also contributes to the entrapment of phytase within core–shell hydrogel beads, supporting the structural stability of phytase as an additional solid barrier.

### 3.3 Thermal stability of phytase

#### 3.3.1 Release profile and secondary structure analysis

**Fig. 6:**
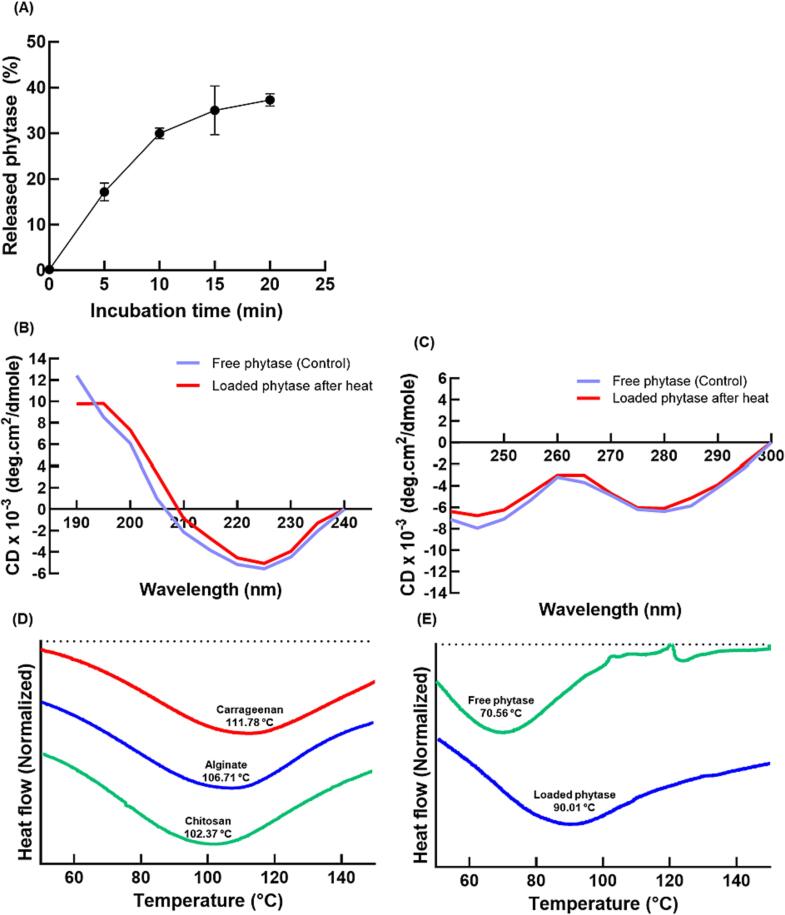
(A) Release profile of phytase from core-shell hydrogel beads during heat treatment at 90 °C for 20 min. (B) Far-UV CD and (C) near-UV CD spectra of free phytase and loaded phytase after heat treatment at 90 °C for 10 min. All samples were incubated in EDTA 0.2 M solution overnight before CD analysis. Thermogram analysis of phytase-loaded core–shell hydrogel beads; (D) The endothermic reaction and melting points of chitosan, alginate, and carrageenan. (E) The endothermic reaction and melting points of free phytase and phytase-loaded core–shell hydrogel beads. All samples were heated from 50 °C to 150 °C.

To confirm the thermal stability, the cumulative release behavior of phytase from the hydrogel beads was investigated after incubation at 90 °C for 20 min (Fig. 6(A)). After 20 min, the amount of phytase that was released was 37.3 ± 1.4 % of the loaded phytase. Notably, the released amount of phytase after heat treatment for 10 min is about 30.0 ± 1.2 %, almost the same as the amount of catalytic activity loss (29.33 %). The low loss of phytase from the core–shell hydrogel bead indicated that the loaded phytase was protected and had good heat resistance.

Circular Dichroism (CD) was used to investigate the effect of heat treatment on the secondary structure of phytase within the core–shell hydrogel bead. There is an observed decrease at 190 nm and an increase at 220 nm when heat treatment destabilizes protein or secondary structures like β-sheet and α-helix (Miles et al., 2021). Specifically, it has been reported that the α-helix content of proteins decreases when the temperature exceeds a protein-dependent specific range (Weng et al., 2023). It has been demonstrated that α-helices play an important role in maintaining the structural conformation of proteins.

To an aqueous suspension of heat-treated core–shell hydrogel beads, EDTA was added to redispersed phytase-loaded beads. The solution was then centrifuged, and the released phytase was analyzed using CD spectroscopy. The results of far-UV CD showed the two expected peaks, 190 nm, and 220 nm, corresponding to the percentage of β-sheets and α-helices (Fig. 6(B)). Compared with raw phytase, the remaining phytase in the beads after heat treatment showed the same result without meaningful change in the pattern of phytase molar ellipticity plotted against wavelength. The lack of substantive change in the CD spectra confirmed that the core–shell hydrogel bead protected the structure of the phytase from conformational changes even after heat treatment.

The overall tertiary structure of the phytase in the core was investigated using near-UV CD spectroscopy (Fig. 6(C)). The results showed slight modifications in the CD spectrum of the protein after heat treatment. Accordingly, the secondary and tertiary structure change of the encapsulated phytase after heating was negligible, once again suggesting that core–shell hydrogel beads could help the stabilization of phytase against heat by increasing the structural stability.

#### 3.3.2 Thermogram analysis

Differential Scanning Calorimetry (DSC) analysis was used to investigate the protective effect of core–shell hydrogel beads based on the thermal behavior of the crystalline phases of each component. All samples showed negative enthalpy variations with endothermic peaks related to the energy absorbed when the temperature was increased from 50 °C to 150 °C. Endothermic peaks are correlated with water loss associated with hydrophilic groups of the polymers, while exothermic peaks are caused by the degradation of the intermolecular interactions due to dehydration and depolymerization processes, most likely a result of partial decarboxylation of the protonated carboxylic groups and other oxidation reactions (Guinesi and Cavalheiro, 2006, Sarmento et al., 2006). A previous report indicated that the denaturation temperature (T_d_) of phytase increased with thermal stability, and the denaturation of the enzyme was irreversible (Garrett et al., 2004).

The melting points of chitosan (102.37 °C), alginate (106.71 °C), and κ-carrageenan (111.78 °C) caused the initial endothermic peak to be broad with a melting temperature (T_m_) higher than 90 °C (Fig. 6(D)). This result indicated the high structural stability of the core–shell hydrogel against heat stress. Furthermore, κ-carrageenan has the highest T_m_ because it can absorb heat based on heat-induced gelation as an endothermic reaction. This property is likely to increase the observed thermal stability of the phytase.

The T_d_ of natural phytase is 70.56 °C (Fig. 6(E)), which is lower than the heat processing temperature (90 °C) and within the range reported for natural phytase (63.7–75.7 °C) (Garrett et al., 2004). The low T_d_ for phytase means that phytase is susceptible to denaturation at food processing temperatures (∼90 °C) (Dersjant-Li et al., 2015). Because of the intermolecular interactions between phytase and the chitosan, the encapsulated phytase in the beads has higher T_d_ (90.01 °C) than raw phytase. Additionally, it is expected that the endotherm of raw phytase after melting involves dissociation and unfolding, whereas the endotherm of encapsulated phytase does not exhibit dissociation behavior ((Guinesi and Cavalheiro, 2006, Sarmento et al., 2006)).

## 4 Conclusions

A core–shell hydrogel bead was fabricated to maintain the catalytic activity of phytase and protect it from heat treatment and proteases. This hydrogel bead has a core composed of a phytase-chitosan complex that stabilizes and protects the phytase and a shell composed of cross-linked alginate-κ-carrageenan that provides heat resistance. The optimized preparation conditions for the core–shell hydrogel bead were found to be 4 w/v % chitosan, 2 w/v % phytase, and 0.2 M calcium chloride. This mixture is then added drop-by-drop to a solution containing a 1:1 ratio of 1 w/v % alginate and 1 w/v % κ-carrageenan. The resulting core–shell hydrogel bead, when compared with free phytase and loaded phytase, maintains 70.67 ± 5.51 % activity compared to natural phytase and its secondary structure even after heat treatment. Furthermore, FTIR and DSC analyses were conducted to identify the mechanism by which the thermal stability of phytase is enhanced. FTIR showed that core–shell hydrogel beads have a strong interaction between chitosan and phytase and crosslinking between alginate and κ-carrageenan. These intermolecular interactions increased the melting point above that of free phytase. Accordingly, this system can help the broad application of phytase with higher structural stability and also be used as the encapsulation system for pharmaceutical enzymes and proteins that have low thermal stability.

## CRediT authorship contribution statement

**Eunhye Yang:** . **Hongmin Dong:** Conceptualization, Investigation, Writing – review & editing. **Waritsara Khongkomolsakul:** Conceptualization, Investigation, Writing – review & editing. **Younas Dadmohammadi:** Conceptualization, Supervision, Writing – review & editing. **Alireza Abbaspourrad:** Conceptualization, Project administration, Supervision, Funding acquisition, Writing – review & editing.

## Declaration of competing interest

The authors declare that they have no known competing financial interests or personal relationships that could have appeared to influence the work reported in this paper.

## Data Availability

Data will be made available on request.
